# Genome-wide association study of primary open angle glaucoma risk and quantitative traits

**Published:** 2012-04-28

**Authors:** Jane Gibson, Helen Griffiths, Gabriella De Salvo, Mick Cole, Aby Jacob, Alex MacLeod, Yit Yang, Geeta Menon, Angela Cree, Sarah Ennis, Andrew Lotery

**Affiliations:** 1 Genetic Epidemiology and Genomic Informatics Group, Human Genetics, Faculty of Medicine, University of Southampton, Southampton General Hospital, Southampton, UK; 2 Clinical Neurosciences Research Grouping, Clinical and Experimental Sciences, Faculty of Medicine, University of Southampton, Southampton General Hospital, Southampton, UK; 3 Southampton Eye Unit, Southampton General Hospital, Southampton, UK; 4 Department of Ophthalmology, Torbay District General Hospital, South Devon Healthcare NHS Foundation Trust, Torbay, UK; 5 Wolverhampton Eye Infirmary, New Cross Hospital, The Royal Wolverhampton Hospitals NHS Trust, Wolverhampton, UK; 6 Ophthalmology Department, Frimley Park Hospital, Frimley Park Hospital NHS Foundation Trust, Guildford, Surrey, UK

## Abstract

**Purpose:**

Primary open angle glaucoma (POAG) is a characteristic optic neuropathy which progresses to irreversible vision loss. Few genes have been detected that influence POAG susceptibility and other genes are therefore likely to be involved. We analyzed carefully characterized POAG cases in a genome-wide association study (GWAS).

**Methods:**

We performed a GWAS in 387 POAG cases using public control data (WTCCC2). We also investigated the quantitative phenotypes, cup:disc ratio (CDR), central corneal thickness (CCT), and intra-ocular pressure (IOP). Promising single nucleotide polymorphisms (SNPs), based on various prioritisation criteria, were genotyped in a cohort of 294 further POAG cases and controls.

**Results:**

We found 2 GWAS significant results in the discovery stage for association, one of which which had multiple evidence in the gene ‘neural precursor cell expressed, developmentally down-regulated 9’ (*NEDD9*; rs11961171, p=8.55E-13) and the second on chromosome 16 with no supporting evidence. Taking into account all the evidence from risk and quantitative trait ocular phenotypes we chose 86 SNPs for replication in an independent sample. Our most significant SNP was not replicated (p=0.59). We found 4 nominally significant results in the replication cohort, but none passed correction for multiple testing. Two of these, for phenotypes CDR (rs4385494, discovery p=4.51x10–5, replication p=0.029) and CCT (rs17128941, discovery p=5.52x10–6, replication=0.027), show the consistent direction of effects between the discovery and replication data. We also assess evidence for previously associated known genes and find evidence for the genes ‘transmembrane and coiled-coil domains 1’ (*TMCO1*) and ‘cyclin-dependent kinase inhibitor 2B’ (*CDKN2B*).

**Conclusions:**

Although we were unable to replicate any novel results for POAG risk, we did replicate two SNPs with consistent effects for CDR and CCT, though they do not withstand correction for multiple testing. There has been a range of publications in the last couple of years identifying POAG risk genes and genes involved in POAG related ocular traits. We found evidence for 3 known genes (*TMCO1*, *CDKN2B*, and S1 RNA binding domain 1 [*SRBD1*]) in this study. Novel rare variants, not detectable by GWAS, but by new methods such as exome sequencing may hold the key to unravelling the remaining contribution of genetics to complex diseases such as POAG.

## Introduction

Primary open angle glaucoma (POAG) is the most common subtype of glaucoma, which can be regarded as a group of diseases with characteristic optic neuropathy that causes a distinctive pattern of progressive visual field loss that could eventually lead to blindness [1]. Several genes known to cause POAG have been identified, such as myocilin (*MYOC*),  optineurin (*OPTN*), WD repeat domain 36 (*WDR36*), and neurotrophin 4 (*NTF4*), though the exact mechanisms by which they are causal remains unclear [2-5]. Conflicting evidence for association has created uncertainty about the importance of *OPTN* and *NTF4* in POAG [6,7] in the general population, though there is evidence that *OPTN* may be specific for normal tension glaucoma (NTG) [3]. These genes are rare causes of the disease, and are detected in families affected by glaucoma, and thus account for few (<10%) POAG cases in total [8]. Association studies detect more common causes of disease and, more recently, several replicated genes have been detected using this method either on a genome-wide scale or by candidate gene analysis. Examples are a variant near the caveolin 1 (*CAV1*) and caveolin 2 (*CAV2*) genes on chromosome 7 [9,10]; transmembrane and coiled-coil domains 1 (*TMCO1*) and CDKN2B antisense RNA 1 (*CDKN2B-AS1*) [11], ELOVL fatty acid elongase 5 (*ELOVL5*), S1 RNA binding domain 1 (*SRBD1*) [12].

There have also been recent successes in identifying genes associated with quantitative ocular traits important in POAG such as intraocular pressure (IOP), vertical cup disc ratio (CDR), optic disc area, and central corneal thickness (CCT). Genes known to be involved with these traits include atonal homolog 7 (*ATOH7*), transforming growth factor, beta receptor III (*TGFBR3*), SIX homeobox 1 (*SIX1*), and caspase recruitment domain family, member 10 (*CARD10*) with optic disc size and vertical cup disc ratio [13-15], as well as zinc finger protein 469 (*ZNF469*), A kinase (PRKA) anchor protein 13 (*AKAP13*), and collagen, type V, alpha 1 (*COL5A1*) with CCT [16-18].

Genome-wide association studies (GWAS) have been performed in a wide range of complex diseases, including diabetes, age-related macular degeneration, Crohn’s disease and bipolar disorder [19,20]. Many common variants have been reproducibly associated with these and many other common diseases. GWAS has therefore been the principal strategy employed, over the last few years, to uncover the genetics of complex traits. We performed a GWAS of risk in 387 POAG patients and 5,830 Wellcome trust case controls consortium (WTCCC2) controls and assessed genetic correlation with quantitative ocular traits in these 387 cases and 50 Southampton controls. We then followed up promising single nucleotide polymorphisms (SNPs) in a further 294 POAG patients.

## Methods

### Patient samples and phenotypes

Three hundred and eighty seven (387) primary open angle patients from a cohort of patients being recruited in Hampshire (UK) were included in this study. Patients were recruited following the tenets of the declaration of Helsinki, informed consent was obtained and the research was approved by the Southampton & South West Hampshire Research Ethics Committee. Patients were all diagnosed as POAG cases and further defined as normal tension glaucoma (NTG) if the average IOP over both eyes ≤21 mmHg, and high tension glaucoma (HTG) if otherwise. All showed visual field loss in at least one eye. A full description of the cohort is given elsewhere [21]. Cases diagnosed as psuedoexfoliation glaucoma and those with a known myocilin mutation were excluded. The replication sample consisted of 294 further POAG cases collected from Southampton, Portsmouth and additional sites on this study in Frimley, Torbay, and Wolverhampton. Myocilin positive cases could not be excluded due to incomplete screening. Descriptions of both the discovery and replication sample are given in Table 1.

**Table 1:** Demographic information on patients and controls of POAG discovery and replication samples.

** Descriptive**	**Discovery sample POAG cases**	**Replication sample POAG cases**	**p-value differences between discovery and replication samples**	**WTCCC2 controls**	**Southampton controls**
No. of subjects	n=387	n=294		n=5380	n=50
**Diagnosis**					
POAG	387	294		NA	0 (0%)
HTG	319(82%)	233(79%)		NA	0 (0%)
NTG	68(18%)	61(21%)	p=0.3†	NA	0 (0%)
**Age in years**					
Mean	75.3	73.1		NA	79
Standard deviation	10.6	10.7	p=0.0077*	NA	4.9
**Gender**					
Female	193	144		2647	27
Male	194	150	p=0.82†	2733	23

This table describes the diagnosis, age and gender of the discovery and replication POAG case and control samples. POAG=primary open-angle glaucoma; HTG=high tension glaucoma, NTG=normal tension glaucoma; p-value *significance of independent samples T- test, †significance of 2×2 χ^2^ test; NA=not available. WTCCC2=Wellcome Trust Case Control Consortium 2 controls –including 1958 British birth cohort and national blood service cohort members. Southampton Controls –have no sign of Glaucoma or Age-related macular degeneration.

The quantitative ocular traits analyzed were; intraocular pressure (IOP) taken as the maximum observed and average value per patient (measured by Goldman applanation tonometry), cup:disc ratio taken as the worst eye and average per patient, and central corneal thickness (CCT) taken as average over both eyes (measured by ultrasound pachymetry- Tomey pachymeter SP-3000; Tomey USA, Phoenix, AZ). Although data were mostly complete for IOP and CDR, fewer data were available for CCT. These ocular traits were also measured in the 50 Southampton controls. Details are given in Table 2.

**Table 2:** Quantitative trait phenotypes for patients and controls of POAG discovery and replication samples.

	**Discovery sample POAG cases (n=387)**		**Replication sample POAG cases (n=294)**		**Southampton control sample (n=50)**	
**Descriptive**	**worst eye**	**average**	**worst eye**	**average**	**worst eye**	**average**
**Highest observed intraocular pressure (IOP)**						
Mean	27.75	25.90	26.7	24.9	15.74	15.53
SD	6.38	5.44	7.04	5.8	3.02	3.00
Minimum	14	14	13	12.5	10	10
Maximum	60	54	58	43	21	21
N	387	387	282	282	50	50
Missing data	0	0	12	12	0	0
**Cup:disc ratio (CDR)**						
Mean	0.78	0.72	0.76	0.71	0.32	0.30
SD	0.14	0.15	0.16	0.15	0.13	0.12
Minimum	0.08	0.08	0	1	0.1	0.1
Maximum	1	1	1	0.975	0.7	0.6
N	387	387	283	283	49	49
Missing data	0	0	11	11	1	1
**Central corneal thickness (CCT) average over both eyes**						
Mean	534		539		552	
SD	34.19		40.5		34.5	
Minimum	417		398		439	
Maximum	624		639		620	
N	191		201		48	
Missing data	196		93		2	

This table gives statistical descriptions of the quantitative ocular traits measured in the discovery and replication POAG cases and the replication controls. No phenotypic information is available for the discovery WTCCC2 controls.

### Genotyping and QC

The discovery sample was genotyped using the Affymetrix SNP 6.0 array (Affymetrix, Santa Clara, CA) and frequencies were compared with the Affymetrix SNP 6.0 array data available for approximately 5,000 WTCCC2 controls, originating from the National Blood Service and the 1948 British birth cohort. The replication sample genotyping was performed using KASPar chemistry.

Quality control steps involved removing cases and SNPs with a high degree of missingness, and removing SNPs with a minor allele frequency less than 5%. We also performed identity by state (IBS) analysis to identify unknown relatives or duplicates and multi-dimensional scaling (MDS) to identify those with differing ethnic backgrounds to the majority of the group (a Caucasian cohort). The WTCCC2 controls clustered tightly together with our cases in the MDS plot, showing that our cases and controls were ethnically compatible (Appendix 1). A total of 387 cases (from 400 genotyped) and 5,380 controls remained for analysis in the discovery sample after these steps.

A Hardy–Weinberg equilibrium (HWE) test (in the WTCCC2 control data) identified SNPs with large deviations (p<0.001) that were excluded from analysis, controlling for possible genotyping errors in the controls. As the cases were genotyped separately, further filtering steps were undertaken to control for possible genotyping error in the cases. See Appendix 2 (supplementary methods) for details. All QC steps were performed using PLINK [22], and 681,552 SNPs remained for analysis. A QQ plot is shown in Appendix 3.

### Statistical analysis

We analyzed the data for risk using allelic χ^2^, and further subdivided our cases into NTG and HTG cases which were independently tested against the WTCCC2 controls. The quantitative ocular traits were analyzed by linear regression, with each SNP tested against each of the traits.

GWA studies are prone to detection of false positives; to ensure that the most promising signals were taken through to the replication stage we applied a ‘clumping’ strategy [22]. Significant SNPs were clumped together as a single association signal if in linkage disequilibrium over a 250 kilobase pair distance. Prioritization for replication was as follows; SNPs with multiple supporting independent clumps in the same region; support from quantitative trait results, as this is not dependent on the WTCCC2 controls; significant SNPs with a p-value ≤10^−6^ with some support in the surrounding region; finally, where clumped results located within a gene, relevant functional data was also taken into consideration.

This multi-pronged approach gave a total of 86 SNPs to test in the replication cohort. All analysis steps were performed using PLINK [22].

## Results

### GWAS results

**Figure 1:**
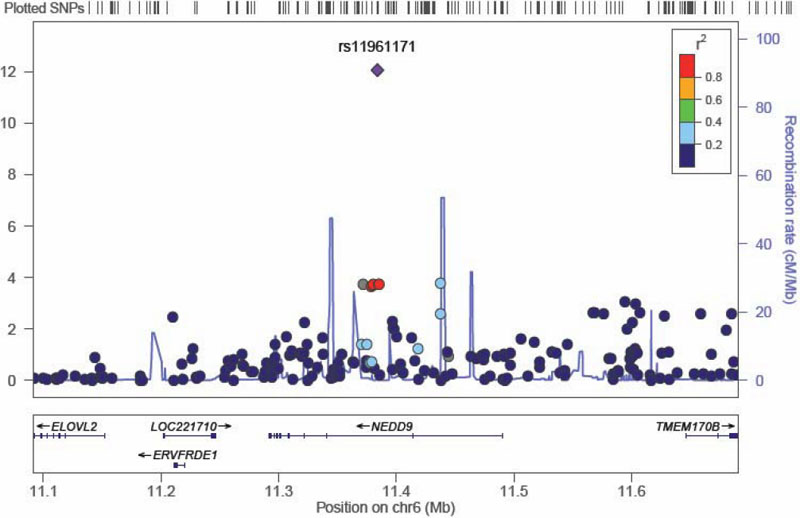
A plot of the most significant region in the discovery sample GWAS. This plot shows the region around the most significant result in the discovery sample GWAS. SNPs are plotted as the -log10 of the p-value. The plot was produced using LocusZoom.

**Figure 2:**
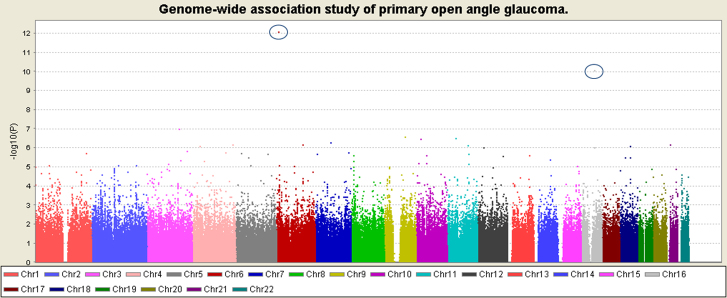
A Manhattan plot of the discovery sample GWAS results. This Manhattan plot shows the results of the discovery sample GWAS for all autosomes. Results are plotted as –log10 of the p-value. The top two results are circled, on chromosome 6 and chromosome 16. No other SNPs reach a stringent genome-wide significance level of 10^-8^. This plot was produced using Haploview.

There were two SNPs with GWAS significance (p<1×10^−8^) in the risk analysis, the most significant was on chromosome 6 (rs11961171, p=8.55±10^−13^) and had 6 SNPs (rs41463745, rs4713332, rs16871186, rs16871188, rs4713335, and rs16871204) in the clump (p≤0.0002) and four other independent SNPs in the surrounding region (Figure 1). The genes nearest to the signal are neural precursor cell expressed, developmentally down-regulated 9 (*NEDD9*), *LOC100129322*, and transmembrane protein 170B (*TMEM170B*). We chose three independent SNPs to genotype in this region in the replication sample. The second GWAS significant SNP was on chromosome 16 (rs924463) with no supporting evidence from the surrounding region suggesting a likely false positive. Figure 2 shows a Manhattan plot of the risk GWAS.

### Quantitative traits

The quantitative ocular traits are summarized in Table 2. As expected for POAG cases the IOP was raised, the CDR increased and the CCT reduced compared to controls. The values were similar in the discovery and replication cases.

### Replication results

For replication 86 SNPs were genotyped in the 294 new cases, 50 controls, as well as the discovery sample allowing data quality checks. The overall concordance rate between the discovery and replication data was >99%. All SNPs passed the HWE test (p>0.001 in controls).

SNPs were analyzed for association to the phenotype which led to their inclusion in the replication cohort. Results for the most significant region in the GWAS and all SNPs which showed significant replication are given in Table 3 and full results are listed in Appendix 4. There were 4 results with a p-value <0.05, none of which pass a multiple testing correction for 86 SNPs. One SNP, located downstream of the gene ‘KH domain containing, RNA binding, signal transduction associated 3’ (*KHDRBS3*), was significant for average CDR, and in a consistent direction to the discovery data. One SNP, located within the gene ‘ubiquitin protein ligase E3 component n-recognin 7’ (*UBR7*), was significant for the phenotype CCT and also had an effect in the same direction as the discovery data. Functional information is lacking for *UBR7*. Ocular trait information had also been collected for the 50 Southampton controls, enabling a similar quantitative analysis in controls for these 2 SNPs. Neither was significant perhaps suggesting that their effects are specific to glaucoma cases, although the control sample size is limited. The final 2 significant SNPs (one for HTG and one for NTG) showed association in the opposite direction to the discovery data, indicating false-positive results.

**Table 3:** Results for the top hit in the POAG GWAS discovery sample, and all nominally significant replication results.

**Phenotype**	**SNP**	**Location**	**Nearest gene(s)**	**Frequency of allele 1 in discovery cases, controls,(allele2)/Qtrait mean direction**	**Frequency of allele 1 in replication cases, controls,(allele2)/Qtrait mean direction**	**Discovery p-value**	**Replication p-value**
**Most significant GWAS region**							
POAG	rs11961171	chr6:11384014	*NEDD9*	A=0.1158, 0.05332 (G)	A=0.04762, 0.06 (G)	8.55E-13	0.5974
POAG	rs967473	chr6:11437409	*NEDD9*	A=0.1899, 0.1407 (G)	A=0.1382, 0.15 (G)	0.000167	0.7538
POAG	rs9366868	chr6:11811486	*C6orf105*	A=0.3329, 0.4098 (G)	A=0.4041, 0.49 (G)	2.59E-05	0.1074
**Significant at replication**							
avCCT	rs17128941	chr14:92754850	*C14orf130 (UBR7)*	T->C increasing CCT	T->C increasing CCT	5.52E-06	0.02743
avCDR	rs4385494	chr8:136919236	*KHDRBS3*	T->G increasing CDR	T->G increasing CDR	4.51E-05	0.02937
HTG	rs4237260	chr9:75931236	*RORB*	C=0.3199, 0.233 (G)	C=0.206, 0.35 (G)	4.98E-07	0.001949*
NTG	rs7785999	chr7:32427787	*PDE1C*, *LSM5*	A=0.2692, 0.1467 (C)	A=0.09483, 0.1939 (C)	9.22E-05	0.02932*

This table gives the results of the most significant SNPs in the discovery sample and all nominally significant (p<0.05) results in the replication sample, including allele frequencies for risk analyses and direction of effect for quantitative trait analyses. *The effect is in opposite directions in the discovery and replication samples.

### Previously published POAG genes

Included in our replication data were 3 SNPs near published POAG risk genes which showed strong evidence for association in our discovery data. SNPs in *SRBD1* and *MTAP* (near *CDKN2B*) both showed strongest evidence for the HTG group, and a SNP in *CDKN2B* which showed strongest evidence in the NTG group. However, these SNPs were not significant in our replication sample (Appendix 4). Table 4 gives a summary of the evidence for published POAG genes, with *SRBD1, CDKN2B*, and *TMCO1* giving the most convincing evidence in our larger discovery sample. The best of several significant SNPs within *SRBD1* is rs11884064 (p=6.7×10^−5^), though nearby rs1657855 is marginally more significant (p=2.69×10^−5^).

**Table 4:** Evidence for published POAG risk and quantitative trait genes.

**Gene (region)**	**phenotype**	**Published SNP**	**Published discovery p-value**	**Reference**	**P-values in our discovery data (where SNP is available)**
*CAV1/CAV2*	POAG	rs4236601	5.0×10^−10^	[9]	Not in our data
*TMCO1*	POAG	rs7518099	4.7×10^−10^	[11]	8.9×10^−4^
*SRBD1*	POAG/NTG	rs3213787	2.5×10^−9^	[12,26]	not in our data. rs2082876 p=0.0005 POAG, p=4.28×10^-5^ HTG, see results section.
*CDKN2B*	POAG	rs10120688	1.4×10^−8^	[11]	1.2×10^-5 ^*evidence also in nearby MTAP
*SIX1*	VCDR/POAG	rs10483727	2.93×10^−10^	[15]	0.3 CDR/0.076 POAG
*ELOVL5*	POAG/NTG	rs735860	4.14×10^−6^	[12]	0.6 POAG/0.7 NTG
*ATOH7*	Disc area	rs3858145 rs1900004 rs690037	6.2×10^−10^ 1.3×10^−10^ 1.5×10^−7^	[14]	We have not tested disc area rs1900004 p=0.07 for CDR. The others not in our data
*CDC7/TGFBR3*	Disc area	rs1192415	7.57×10^−17^ (meta p-value)	[13]	not in our data
*CARD10*	Disc area	rs9607469	2.73×10^−12^ (meta p-value)	[13]	0.79 for CDR
*ZNF469*	CCT	rs12447690	2.87×10^−8^	[16]	not in our data
*COL5A1*	CCT	rs1536482	7.1×10^−8^	[17]	not in our data
*AKAP13*	CCT	rs6496932	1.4×10^−8^	[17]	0.23
*COL8A2*	CCT	rs274754	0.018	[27]	not in our data
*AVGR8*	CCT	rs1034200	3.5×10^−9^	[17]	not in our data

This table lists genes or regions previously published to be associated with POAG or related ocular traits. Published SNPs and p-values are given along with relevant p-values in our discovery sample (where data were available).

We found no association evidence for SNPs within *MYOC*, as expected, since patients with *MYOC* mutations were excluded. Also *MYOC* mutations along with *WDR36* and *OPTN* are rare causes of POAG and thus not expected to be detected by GWAS. We found some very weak evidence in *OPTN* (p=0.02), but none in *WDR36*.

## Discussion

We report two SNPs highly significant in our discovery data for the phenotypes CCT and CDR. Both have positive replication data, neither of which withstands a multiple testing correction, but both show direction of effects consistent with the discovery data. The most significant SNP in the POAG risk analysis is located in *NEDD9*. There was strong evidence from surrounding SNPs and *NEDD9* appears an excellent candidate as it has been shown to be increased in trabecular meshwork cells [23], however, none of the 3 SNPs chosen for follow-up in the *NEDD9* region were significant in the replication study. As GWAS studies are prone to type one (false positive) error, we chose to follow-up the SNPs with most evidence, based on a variety of criteria, rather than simply single p-values. We believe our selection criteria enhanced our likelihood of successful replication, but it is possible we may have excluded some real associations.

Several genes of moderate effect have been detected by GWAS, including variants near *CAV1* and *CAV2* [9] and *TMCO1* and *CDKN2B* [11]. We assessed evidence for a list of well replicated published genes in our GWAS (Table 4) and found the genes *SRBD1*, *CDKN2B*, and *TMCO1* had the most convincing evidence of association in our data. Interestingly, the best SNP within *SRBD1* is rs11884064 (p=6.7×10^−5^), though nearby rs1657855 is marginally more significant (p=2.69×10^−5^) and located upstream of *SRBD1* and nearer the *SIX3* and *SIX2* genes which play roles in eye development.

Few GWAS significant signals have been detected for POAG, even in studies with very large sample size [9]. It seems that the remaining POAG heritability may be accounted for, by rare variants across multiple genes that all contribute to genetic risk and these are not amenable to discovery using genome-wide association methodology. GWAS are aimed at identifying common SNPs with allele frequency of >5% based on the common variant-common disease hypothesis of disease pathogenesis. These genetic polymorphisms may individually only modestly increase the risk of disease. However, there is increasing evidence that accumulation of rare variants may have a larger impact on complex disease than first thought, and may be responsible for the as yet unaccounted for genetic contribution to some common complex diseases. Recent identification of a rare penetrant variant in AMD as an example [24]. Such variation will be detectable by new methods in next generation sequencing which allows genetic variation to be cataloged for all genic regions or the whole human genome. The genetic variants accounting for the remaining heritability of POAG may be more suited to detection by this type of study. There are also several ocular traits (sub-phenotypes), relevant to POAG diagnosis and disease progression, which have been associated with genetic variation in the population and in the POAG subgroup. There appears to be a complex interplay between genes involved in eye development and maintenance, which are also involved in susceptibility to the common form of glaucoma (POAG) [25].

## Appendix 1. Multi-dimensional Scaling (MDS) plot of ethnicity clustering.

POAG cases and WTCCC2 controls are plotted along with HapMap phase3 data. Ethnic groups cluster together and the POAG and WTCCC2 samples cluster together with the HapMap CEU (Utah residents with ancestry from northern and western Europe) and the TSI (Toscans in Italy) samples. The red squares with a white cross labelled ‘Remove’ are samples that fell away from the main cluster and were removed from analysis. To access the data, click or select the words “Appendix 1.” This will initiate the download of a compressed (pdf) archive that contains the file.

## Appendix 2. Supplementary methods.

To access the data, click or select the words “Appendix 2.” This will initiate the download of a compressed (pdf) archive that contains the file.

## Appendix 3. QQ plot of autosomes.

To access the data, click or select the words “Appendix 3.” This will initiate the download of a compressed (pdf) archive that contains the file.

## Appendix 4. All SNPs tested in replication cohort with p-values and frequencies for discovery and replication data, and direction of effect for the quantitative traits.

Grey cells highlight known genes and significant replication results. To access the data, click or select the words “Appendix 4.” This will initiate the download of a compressed (pdf) archive that contains the file.
